# Levels of elastic resilin modulate leg stiffness but not elasticity in *Drosophila*

**DOI:** 10.1016/j.isci.2026.116404

**Published:** 2026-06-17

**Authors:** Sarah Oeftger, Bernard Moussian, Fritz-Olaf Lehmann

**Affiliations:** 1 Department of Animal Physiology, Institute of Biosciences, University of Rostock, Albert-Einstein-Street 3, 18059 Rostock, Germany; 2 Institute Sophia Agrobiotech, University of Nice Sophia Antipolis, 38 Av. Emile Henriot, 06000 Nice, France

**Keywords:** animal physiology, genetic engineering, animal biotechnology, biomechanics

## Abstract

Resilin is a highly effective elastic component of the insect cuticle, saving energy, providing flexibility, and protecting chitinous structures against structural damage. In the locomotor system of *Drosophila*, the protein occurs patchy at various locations of the leg. Here, we genetically enhanced and also eliminated resilin by 34% and −69%, respectively, in the long tendon of *Drosophila* and quantified leg stiffness and elasticity in a work-loop-based biomechanical testing assay during leg movement. Resilin content little alters leg articulation and posture angles during force loading, while elevated resilin expression enhances leg stiffness 3.9-fold compared to resilin knockout and 2.2-fold compared to *wild-type* flies. We also found that leg elasticity is unaffected by resilin and discuss our findings with respect to material properties and active neuromuscular control. Collectively, our data fuel future research on resilin-dependent changes in locomotor capacity, efficiency, and energetic costs for leg movement in the behaving insect.

## Highlights

• Resilin is a highly effective elastic component of the insect cuticle
• The protein occurs patchy in leg tendons of fruit flies
• Levels of resilin alter overall leg stiffness in living flies
• Leg elasticity is unaffected by resilin within a force loading-unloading cycle

## Graphical abstract

**Figure:**
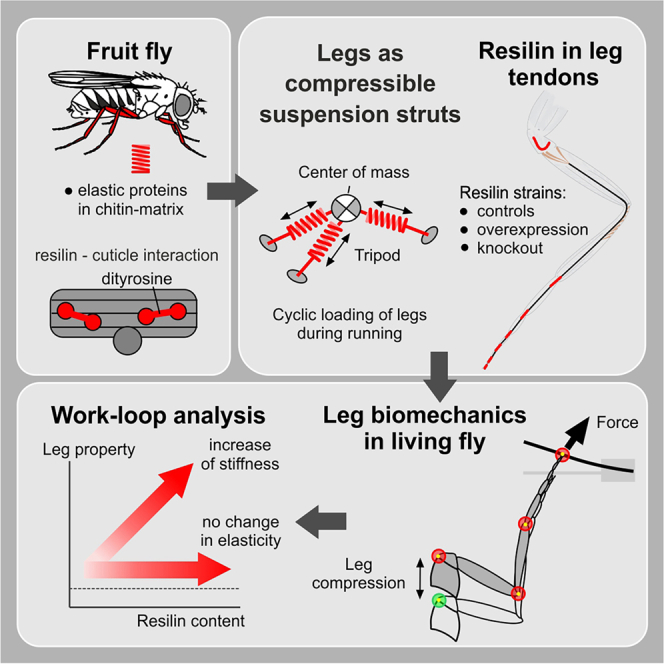

## Introduction

Rubber-like elasticity is a property of many proteins in animals that allows movements of tissues without disruption, while saving deformation energy.^1^ Elasticity resides in many proteins, such as polypentapeptides of spider silk, abductin and collagen in bivalves, and elastin of vertebrate muscle tendons, as well as in resilin as part of the composite material in the chitin matrix of arthropods and crustacean cuticle.^1^^,^^2^^,^^3^ In insects, the elastic composites of chitin serve various functions, including the storage of kinetic energy, absorption of mechanical shocks, prevention of cuticle crack propagation, and as mechanical antagonists for muscle action.^4^ Suggested categories for the function of elastic proteins include power amplifiers, elastic energy stores during running and wing flapping, return springs, smoothing flows, cushioning impacts, and locomotor force control.^5^^,^^6^ Thus, elasticity is essential in many animals and determines the animal’s fitness, survival, and adaptability.^7^

Pure resilin has the smallest mechanical damping of all bioelastomers amounting to less than 0.4% energy loss during length changes.^3^ It may deform 20%–40% of its length without plastic deformation, has 92%–97% resilience, and thus withstands ∼300 million lengthening cycles without fatigue.^6^^,^^8^^,^^9^^,^^10^^,^^11^^,^^12^ Resilin, for example, provides flexibility to the internal rod that supports the honeybee tongue,^13^ avoids damage and hemolymph loss of the spermalege of female bed bugs,^13^ determines the stiffness and thus attachment force of the leg’s attachment pads in the silk moth,^14^ and allows elastic energy storage in the wing hinges of locusts, dragonflies, beetles, and fruit flies during flight.^11^^,^^15^^,^^16^^,^^17^^,^^18^ Extensive research has been devoted to the function of resilin during running and jumping of insects. Resilin appears in leg structures of many species such as cockroaches, locusts, fleas, froghopper insects, and fruit flies.^9^^,^^17^^,^^19^^,^^20^^,^^21^^,^^22^ The role for terrestrial locomotion, however, is unclear as resilin is only expressed locally in small amounts. Recent work on the locust leg emphasized the role of resilin for reducing damage to tissues and that a decrease in resilin concentration in the locus leg results in both a 15% decrease in take-off velocity and jump distance, at the cost of an increase in leg fractures.^12^ However, despite previous findings, biomechanical measurements on resilin-chitin structures in living animals are rare and often limited to relatively basic behavioral testing.^12^^,^^14^ Besides, a major problem is that in many animals resilin cannot be removed from the chitin matrix or overexpressed for comparison in a control experiment.

Recent studies on the genetics of resilin in the fruit fly *Drosophila melanogaster* provide more options to understand the functional role of resilin for locomotion.^5^^,^^17^^,^^23^ Typically, resilin never occurs in the insect exoskeleton as a pure substance but rather in the form of a composite material, mixed with other proteins. Most studies on the distribution of resilin in the insect cuticle largely rely on imaging of the fluorescent properties of the interprotein dityrosine (DT) bounds that account for the polymerization of resilin monomers.^24^^,^^25^ Direct localization of both, the resilin monomer pro-resilin and the potential elastic protein cpr56f within the resilin matrix, was successful in the fruit fly *Drosophila melanogaster* using fluorescent-tagged proteins that were genetically coupled to pro-resilin.^17^ In this animal, resilin mainly occurs in the proboscis, wing hinges, legs, spiracles of the tracheal system, and at quite unanticipated locations of the cuticle such as the tracheal endings.^17^

In *Drosophila* legs, pro-resilin and its related cpr56f locally occur at the trochanter, the tibio-tarsal joint, and along the tarsal joint transitions.^17^ However, previous research left open the answer to the question whether pro-resilin is located as a component in the outer cuticle or resides inside the leg and thus in sclerites, muscles, and tendons. In general, insects have cuticular apodemes that act as muscle tendons in vertebrates. Besides its role for active movements during walking and jumping, the tension of leg tendons (apodemes) and muscles is essential for leg posture and stability, and thus for body posture of the sitting fly. A major component that is responsible for leg function is the long leg tendon (pretarsal apodeme, retractor unguis apodeme^26^). The tendon is controlled by long tendon muscles in the femur (retractor unguis muscle 1) and tibia (retractor unguis muscle 2) and runs ventral through the femoro-tibial joint and up to the end of the fifth tarsal segment where it connects to the unguitractor plate.^27^ It directs and helps to stabilize all joints distal to the femur, including the tarsal segments that lack muscles, during jumping and grasping.^27^ The pretarsus only has a depressor muscle, and levitation is thought to result from elasticity of its basal parts as shown in beetles and stick insects.^26^^,^^28^^,^^29^ Muscle tendons in *Drosophila* mainly consist of the rigid polysaccharide protein chitin, which allows only little change in tendon length and provides little elasticity.^30^^,^^31^^,^^32^ Superficially, these properties run counter to the suggested behavior of tendons to cope with length changes and bending stress during joint flexion and to store elastic potential energy during walking and jumping.^30^^,^^31^^,^^32^

**Figure 1:**
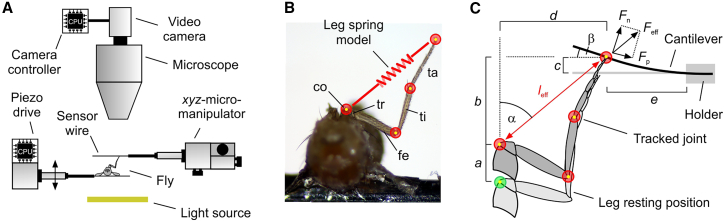
Biomechanical assay for testing leg stiffness and elasticity in fruit flies (A) Experimental setup. The fly is tethered to a piezo-driven micromanipulator that moves the animal with a single leg against a force sensor (tungsten wire). The wire is mounted to a manually driven *xyz*-micromanipulator. The open end of the wire allows the wire to bend under load. Leg position and wire bending are recorded by a video camera mounted to a dissecting microscope. (B) Measurement principle and close-up of the fly leg and force sensor. Red dots indicate leg positions tracked in each video frame for analysis of stiffness and leg segment angles. The leg is modeled as a compressible suspension strut connecting the coxa with the pretarsus. co, coxa; tr, trochanter; fe, femur; ti, tibia; ta, tarsi. (C) Body displacement and forces acting on the sensor wire. A crossbar at the tarsal attachment point hinders the leg to move sideways. l_eff_, effective leg length; α, leg extension angle; β, wire bending angle; a, body displacement; b, vertical distance; c, wire displacement; d, leg extension; e, sensor length (40 mm); F_n_, sensor bending force; F_p_, parallel force; F_eff_, effective force on leg. Drawings are not to scale and wire bending is exaggerated.

Our study approaches the role of resilin for leg function in *Drosophila* using genetically modified strains with reinforced expression rates of *pro-resilin* (*res*^*+*^*-*Venus, *res*^*+*^*-*GFP), and also *pro-resilin*-knockout strains (*res*^*–*^). This genetic approach is combined with a histological survey of the distribution of resilin with focus on the long tendon of the fly’s middle leg, biomechanical testing of leg flexibility and stiffness in living animals, and an analysis of the loss of elasticity during a full leg force loading-unloading cycle (Figure 1). For this investigation, we adopted the work-loop concept that was originally developed to study skeletal and cardiac muscle contraction properties.^33^^,^^34^ Our data eventually show that resilin fluorescence that is visible on the outside of the leg largely stems from resilin patches of leg tendons inside the leg and that overexpressed rates of *pro-resilin* reinforce the leg’s overall stiffness. Most noteworthy, our study also shows that leg elasticity is independent of the leg’s resilin content.

## Results and discussion

### Verification of *pro-resilin* expression

**Figure 2:**
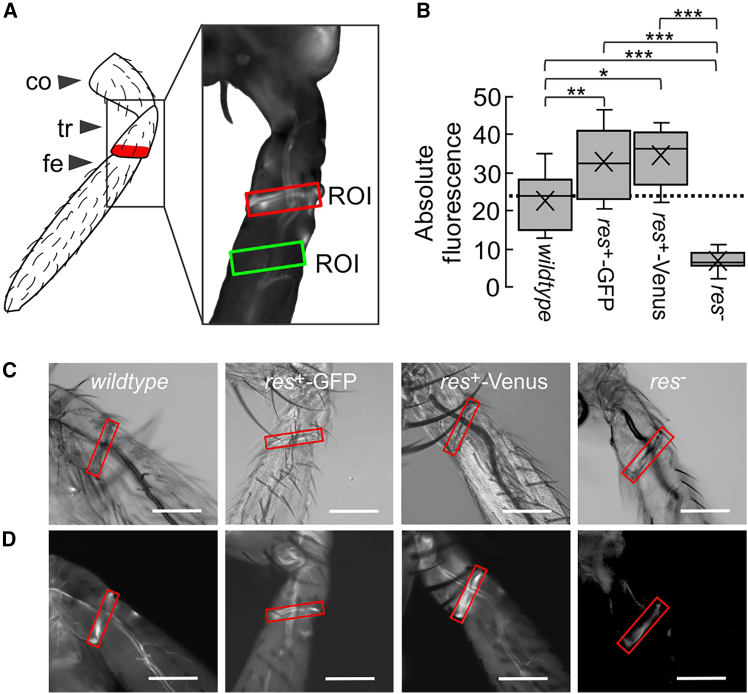
Autofluorescence by resilin tyrosine bonds in *wild-type* and transgene flies (A and B) The measurements validate the activation of genetically inserted *pro-resilin* gene copies. Absolute fluorescence in the *Drosophila* middle leg is shown in (B) for the tested strains including (C) representative bright-field and (D) DAPI-filtered images of single animals. These values convert into relative fluorescence as follows: *wild-type* Canton S, 100%; *res*^*+*^*-*GFP*,* 131%; *res*^*+*^*-*Venus*,* 134%; *resilin*-knockout *res*^*–*^*,* 31%. Fluorescence was measured using DAPI filter in a resilin-rich region (ROI [region of interest]) at the trochanter (red, A, C, D) and corrected for autofluorescence and pigmentation of the cuticle measured in a resilin-free region (green, A). Fluorescence is quantified based on tyrosine bonds that crosslink 20%–25% of pro-resilin molecules (see main text for details). Data are represented as boxplot showing interquartile range (25th–75th percentiles) with whiskers extending to the 5th and 95th percentiles. Medians, horizontal line; means, cross. Dashed line indicates median of *wild-type* flies. co, coxa; tr, trochanter; fe, femur; ∗∗∗*t* test *p* < 0.001, ∗∗*p* < 0.01, ∗*p* < 0.05. *n* = 10 animals of each strain. Scale bars, 100 μm in (C and D).

**Figure 3:**
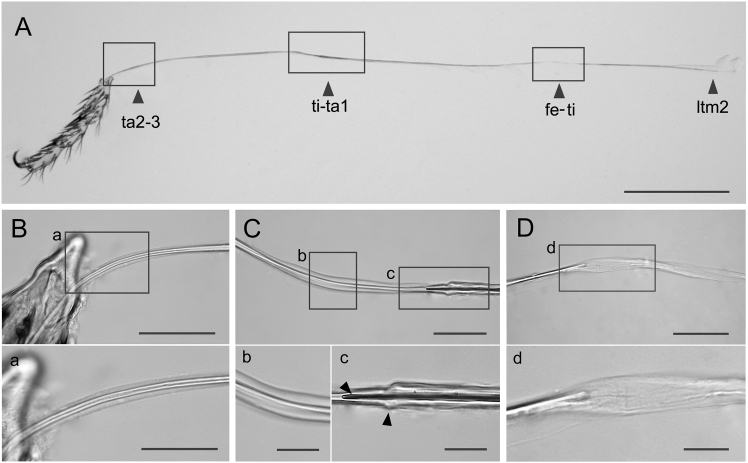
Structure of the leg’s long tendon (pretarsal apodeme, retractor unguis apodeme) in *wild-type Drosophila* visualized by bright-field phase-contrast microscopy (A) Isolated tendon with third to fifth tarsal segments attached. Boxes indicate the position of enlarged pictures shown in (B–D). ta2-3, transition between second and third tarsus; ti-ta1, transition between first tarsus and tibia (tibio-tarsal joint); fe-ti, transition between femur and tibia (femoro-tibial joint); ltm2, long tendon muscle 2 (retractor unguis muscle 2). (B–D) Magnified tendon structure at leg segment joints as shown in (A). Boxes are region of interests, and enlarged image sections are shown in a–d. Arrows in c indicate the sclerotized central rod of the tendon (upper arrow) and its local broadening (lower arrow). Scale bars: 200 μm in (A), 50 μm in (B), 25 μm in (a, C, and D), and 10 μm in (b, c, and d).

**Figure 4:**
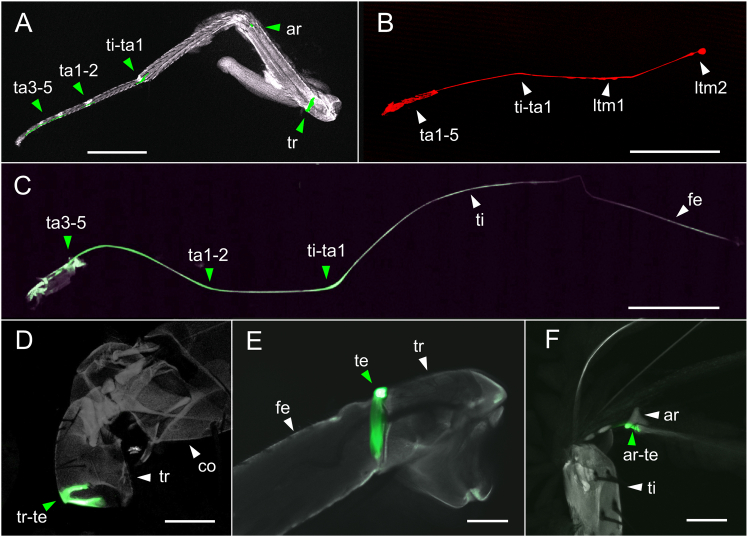
Cuticle autofluorescence, GFP-fluorescence, and chitin staining of the *Drosophila* middle leg (A) Imaging of cuticle autofluorescence in a *res*^*+*^*-*GFP fly (white) superimposed by GFP-fluorescence (green) indicates the expression of *pro-resilin*. Pro-resilin occurs in tarsal segments, femur, and close to the femoro-trochanteral joint. (B) Direct Red 23 staining of the chitin matrix (red) in the long tendon of a *wild-type* fly. (C) Chitin autofluorescence (white) superimposed with GFP-fluorescence (green) of an isolated long tendon from a *res*^*+*^*-*GFP fly. Note that GFP-fluorescence of the tendon coincides with fluorescence of the intact leg as shown in (A), suggesting that resilin is located in tendons and not in the outer cuticle. (D and E) Close-up of the resilin-containing horseshoe-shaped tendons of femur depressor and femur retractor muscles in the trochanter. Dorsal-lateral view in (D) and lateral view on the middle leg in (E). (F) Resilin at the tendon connecting the arculum with the tibia extensor muscle. Sensory tendons between arculum and femoral chordotonal organ are not visible. Images are captured by laser scanning microscopy and appropriate software filter settings for chitin, GFP, and Direct Red 23. ta1-2, transition between first and second tarsus; ta3-5, transitions between third and fifth tarsus; ta1-5, first to fifth tarsus; ar, arculum; co, coxa; te, tendon; ti, tibia; fe, femur; tr, trochanter; ltm1, long tendon muscle 1 (retractor unguis muscle 1). White and green arrows indicate autofluorescence and GFP-fluorescence, respectively. See legend to Figure 3 for more abbreviations. Scale bars: 100 μm in (A), 500 μm in (B), 200 μm in (C), 75 μm in (D), and 50 μm in (E and F).

**Figure 5:**
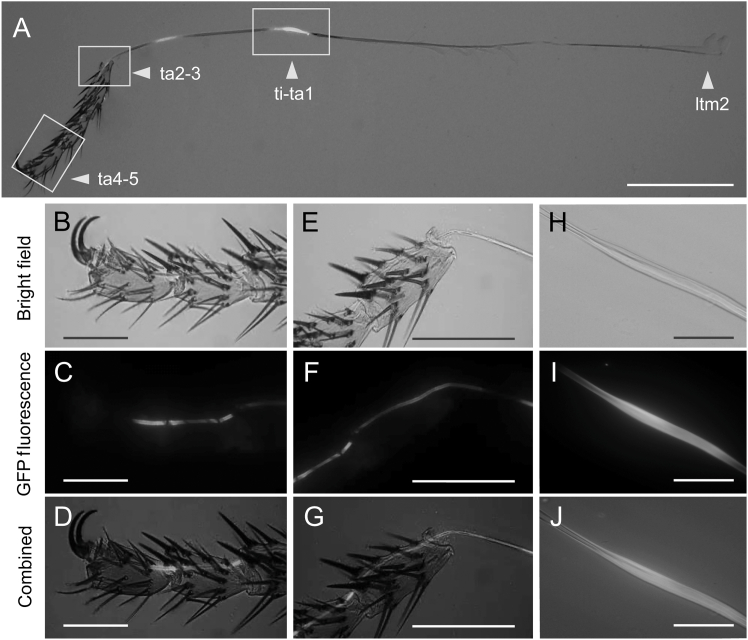
Bright-field imaging and GFP-fluorescence of the long tendon from a *Drosophila* middle leg (A) Superimposed bright-field and GFP imaging of the long tendon measured in a single *res*^*+*^*-*GFP animal. Region-of-interests are enlarged in the pictures below. (B, E, and H) Region of interests as shown in (A) captured by bright-field microscopy. (C, F, and I) Same image sections as in (B, E ,and H) but GFP-fluorescence. (D, G, and J) Bright-field and GFP-fluorescence images superimposed. Images are captured by an epi-fluorescence microscope and appropriate optical filters. For abbreviations see legends to Figures 3 and 4. Scale bars: 200 μm in (A) and 50 μm in (B–J).

We verified expression rates of *pro-resilin* in genetically modified *Drosophila* strains (*cf*. “method details” section) from fluorescence of the tyrosine bonds in the synthesized resilin.^17^ Approximately 20%–25% of pro-resilin molecules are thought to be crosslinked.^11^^,^^35^ For this control, we quantified the fluorescence signal of a resilin-rich leg structure that appears as a small highly visible band at the femoro-trochanteral joint in *Drosophila* (red region of interest [ROI], Figure 2A). This band has previously been described, and its fluorescence likely results from resilin inside tendons of trochanter muscles.^17^ The reason that we used the fluorescence of the trochanter tendons for genetic validation was its better visibility compared to the small patches of resilin in the long tendon (Figures 3, 4, and 5; see also Data S1, supplemental information). To consider chitin autofluorescence, we scored fluorescence of the cuticle at the femur in a resilin-free region next to the joint (green ROI, Figure 2A) and subtracted this value from the signal at the joint in each tested fly. Figure 2B shows a 30.7% increase in resilin fluorescence in *res*^*+*^*-*GFP flies, a 34.1% increase in *res*^*+*^*-*Venus, and a 69.3% decrease in *res*^*–*^ compared to *wild-type* flies (*n* = 10 flies each). Fluorescence in all transgene flies is significantly different from *wild-type* (Shapiro-Wilk test, *p* > 0.05; 2-sided unpaired *t* test, *p* < 0.02), and both *pro-resilin* overexpression strains (*res*^*+*^*-*GFP, *res*^*+*^*-*Venus) are different from the knockout strain (*res*^*–*^, *p* < 0.001), while fluorescence of *res*^*+*^*-*GFP and *res*^*+*^*-*Venus strains is similar (*p* = 0.44). It should be noted that compared to *res*^*+*^*-*GFP flies, UAS *res*^*+*^*-*Venus is ubiquitously expressed in the fly by the *daughterless* (da) promoter. Potentially, this could result in the production of resilin even in tissues, such as epidermal cells, that do not naturally contain resilin. However, previous work demonstrated that expressing a C-terminally Venus-tagged *pro-resilin* in the epidermis does not lead to an increase in resilin formation in this tissue.^17^ The latter was shown by monitoring auto-fluorescence of DT bonds. No DT signal was detected in the ectopic *pro-resilin*-Venus locations indicating that only tissues naturally expressing pro-resilin are competent to initiate DT formation. A likely explanation for this finding is that DT formation requires the dual oxidase (Duox) that is necessarily part of the genetic program in cells naturally synthesizing resilin, but not other cells. Moreover, if resilin would have been synthesized, for example, in the cuticle, this should be visible as an increase of background fluorescence within the control ROI in Figure 2A (green ROI). ROI background fluorescence of the four tested strains is shown in the supplemental information (Data S2). ANOVA indicates that *wild-type*, *res*^*+*^*-*Venus, and *res*^*+*^*-*GFP strains have similar auto-fluorescence (*p* > 0.36; measured with DAPI filterset to detect DT bonds of resilin). Thus, there is no evidence that DTs are formed in the cuticle in excess of the concentration found in *wild-type* flies. The latter finding is consistent with the significant decrease of cuticle fluorescence (*p* < 0.001) in the *pro-resilin*-knockout strain. It should also be noted that an overexpression of *pro-resilin* does not automatically result in an increase of resilin polymerization. If the cross-linking system is saturated, *res*^*+*^ animals would accumulate pro-resilin in the extracellular matrix without adopting the mechanical properties of polymerized resilin.

### Structure of the long tendon

Figure 3 shows details of the structure of the leg’s long tendon in *wild-type Drosophila*. The data confirm previous findings in insects that the tendon runs from its attachment site at the long tendon muscle 2 ([ltm2], Figure 3A) in the proximal femur through the entire leg down to the fifth tarsus.^27^ Muscle attachment sites of long tendon muscles in the tibia and femur appear as local swellings near single muscle fibers (Figures 3 and 4). In general, the images suggest that the tendon is not homogenous but a multi-layer structure, consisting of differently sclerotized internal components. Tendon structure may vary at the joints between leg segments, which is highlighted in Figure 3B for the transition between second and third tarsi, in Figure 3C for the joint between tibia and first tarsus, and in Figure 3D for the joint between femur and tibia. The transition from tibia to the first tarsus, for example, is characterized by the local appearance of a sclerotized central rod that is accompanied by a larger diameter of the tendon (Figure 3C). This pronounced structure is missing in the proximal sections of the tendon and also near the femoro-tibial joint. At the femoro-tibial joint, tendon diameter expands and the tendon appears to be fibrous.

For comparison, similar structures have been reported for the long tendon in other insects such as tenebrionid beetles.^26^ The latter study also reported an increased flattening and sclerotization of the long tendon at the muscle insertion points that potentially helps to redirect muscle force to the tendon. In *Drosophila*, both structures are visible at the joint between tibia and first tarsus (Figure 3C) and also to some degree at the femoro-tibial joint (Figure 3D). However, we did not find local thinning of the tendon at the leg joints as reported for beetles.^26^ The mean tendon diameter in *wild-type* flies is 3.25 ± 0.15 μm (*n* = 10 flies), at an average tendon variance of 1.88 ± 0.05 μm (width of Gaussian fit curve to all tendon diameters; *R*^2^, 0.94 ± 0.01). Mean tendon diameter in *res*^*+*^*-*GFP strains tends to be 3.6% larger than in *wild-type* flies (3.37 ± 0.13 μm with 2.43 ± 0.47 μm mean error, *n* = 10 flies; *R*^2^, 0.91 ± 0.02; unpaired *t*-test, *p* > 0.07). We omitted this small difference for tendon stress calculations and used the tendon diameter of *wild-type* flies for all tested strains. The mean leg diameter (outer cuticle) of femur, tibia, and tarsi were also not significantly different among the tested strains (Data S3, supplemental information).

### Distribution of GFP-tagged pro-resilin

Imaging of GFP-fluorescence in *res*^*+*^*-*GFP animals superficially locates resilin in the outer cuticle of the leg.^17^ Figure 4A suggests that pro-resilin, and thus resilin, is mainly expressed in the tarsal segments, the tibio-tarsal joint, and as a pronounced band near the femoro-trochanteral joint (*cf.* Figure 2). However, as previous research on other arthropods reported resilin also in muscle tendons,^12^^,^^20^^,^^36^ we considered the possibility that at least part of the above GFP-fluorescence stems from pro-resilin inside leg tendons. Figure 4C shows autofluorescence of the long tendon (white) superimposed with GFP-fluorescence (green) of the isolated tendon. We found that GFP-fluorescence occurs exclusively along the tarsal segments, near the tibio-tarsal joint but not in the tibia and the femur. The pronounced fluorescence at the trochanter is not due to resilin of the long tendon but likely stems from the tendons of the femur depressor and femur reductor muscles inside the trochanter.^27^ We found no visible GFP-fluorescence in other leg tendons such as in tendons of the tibia levator and depressor muscles inside the femur and tarsus levator and depressor muscles inside the tibia. Chitin-specific staining of the long tendon with Direct Red 23 suggests that most of the autofluorescence in Figure 4C is due to chitin. The local changes in dye intensity might result from different diameters of the tendon, and the local swellings coincide with the attachment sites of the long tendon muscles 1 (ltm1) and 2 (ltm2). Altogether, the availability of chitin in the tendon supports the assumption that chitin and resilin locally form a composite material for force transfer between the femur, tibia, and tarsi.^5^

Combined imaging of tendon structure (bright-field microscopy) and GFP-fluorescence in the long tendon of *res*^*+*^*-*GFP strain allows us to locate pro-resilin in greater detail. Figure 5 shows local GFP-fluorescence at the tibio-tarsal joint, along larger section of the tarsi, but not at the tip of the fifth tarsal segment holding the claws and pulvilli. Figures 4 and 5 both suggest that the fluorescence at the tarsal segments is due to resilin in the long tendon and that it does not reside in elastic cuticle structures. This distribution pattern may not support the idea that resilin is key for elastic antagonistic movements of the tarsi as suggested for beetles, stick insects, cockroach, and locust legs.^9^^,^^21^^,^^26^^,^^28^ Instead, resilin in *Drosophila* might help to adjust tension and length of the long tendon at leg joints but might not be responsible for the production of forces needed to extend tarsal segments during jumping.

### Resilin-dependent leg folding angles

**Figure 6:**
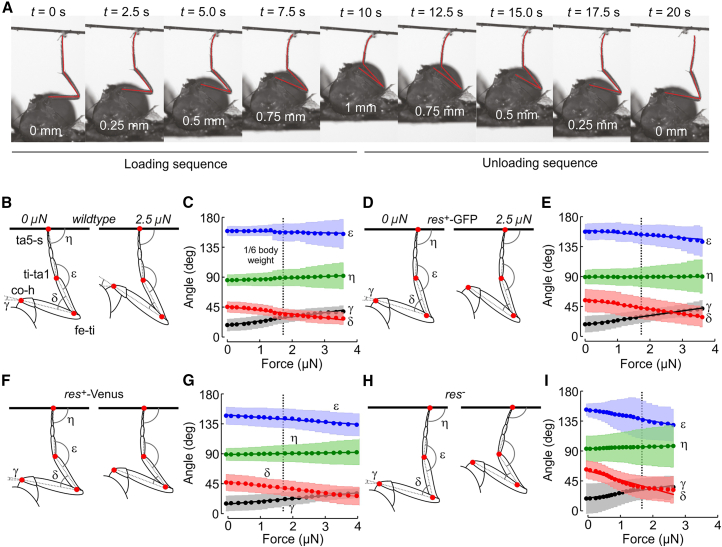
Posture changes of *Drosophila* middle legs during force loading (A) Video images showing folding angles of the left middle leg during force loading sequence (0*–*1.0 mm displacement, 0–10 s) and unloading sequence (10–20 s) of a single *wild-type* fly. Leg compression and expansion occurred in steps of 0.05-mm body displacement with 0.5-s pause between each step. Sequence shows only every fifth step of the piezo-driven movement. Images are centered about the sensor wire. (B, D, F, and H) Leg angles at two force loadings as shown in the figures. (Left) Leg posture without force loading but with tarsal contact on the sensor wire (thick black line, 0 μN). (Right) Leg posture during loading with ∼1.5-fold the force of a sitting fly (2.5 μN). (C, E, G, and I) Change in leg angles in response to increasing force loading. Vertical dashed lines indicate approximately the force the leg experiences in a sitting fly. Lines connecting data points are linear regression fits to the mean values. Data S5, supplemental information, shows number of tested flies and the outcome of linear regression fit analysis. γ*,* angle of coxo-trochanteral joint; δ, angle of femoro-tibial joint; ε, angle of tibio-tarsal joint; η, angle of end of tarsus and substrate. Data are represented as mean ± standard deviation (colored area). *n* = 10 *wild-type*, *n* = 11 *res*^*+*^-GFP, *n* = 12 *res*^*+*^-Venus, and *n* = 12 *res*^*-*^ flies.

A main goal of this study is to attribute resilin content of the *Drosophila* leg to leg biomechanics and structural stiffness. In principle, resilin might alter leg stiffness in two ways: first, due to changes of the mechanical properties of muscle tendons and/or the chitinous exoskeleton and second, by changing control structures containing resilin such as the *arculum*, which is a mechanical structure decomposing movement of the femoro-tibial joint into orthogonal force components for receptors of the femoral chordotonal organ (FCO). In the latter case, resilin could alter leg angles via neural feedback of strain-stress receptors inside the femur.^37^ In a first step, we thus mapped the angles between leg segments during a complete force loading-unloading cycle by tracking leg joints and the position of the fifth tarsus from the recorded videos (Figure 6). For these measurements we developed a biomechanical testing assay, in which a single fly leg was moved in small steps of displacement by a piezo drive against a force sensor wire. The deflection of the sensor wire and the position of the leg joints were recorded by video and analyzed after the experiment (Figure 1A, *cf.* “method details”). The image sequence in Figure 6A shows leg folding of a single *wild-type* fly, and Figures 6B–6I show the means of four leg angles depending on loading force in all tested strains. We did not attempt to estimate angular changes between tarsal segments because these changes were negligible.

We found that in all tested fly strains, leg angles significantly change with changing load (linear regression fit to mean angles, *p*_*slope*_ < 0.001) except for the regression slope of tibio-tarsal angle ε (ti-ta1, Figures 3 and 4) in *res*^*+*^*-*GFP flies (*p* > 0.05). Moreover, the mean elevated regression coefficient (*R*^2^ = 0.81 ± 0.11, *n* = 30 regression coefficients, mean ± standard deviation; Data S5, supplemental information) suggests that all angles linearly change with increasing force. The coxo-trochanteral angle γ slightly increases with increasing loading with values ranging from 1.81° μN^−1^ in *act5C-*cas9 strains to 5.99° μN^−1^ in *wild-type* flies. The femoro-tibial angle δ slightly decreases by minimum −3.32° μN^−1^ in *res*^*+*^*-*GFP flies to maximum −5.33° μN^−1^ in *act5C-*cas9 controls, and the rate of change of the tibio-tarsal angle ε ranges from −0.31° μN^−1^ in *wild type* to −7.63° μN^−1^ in *act5C-*cas9. By contrast, the angle between tarsus and substrate η is largely independent of loading and ranges from −1.62° μN^−1^ in *R104* to 1.61° μN^−1^ in *act5C-*cas9 (Figures 6B–6I).

To statistically test for differences among the six control and pro-resilin-modified strains, we conducted ANOVA analysis with Tukey post hoc test (*p* > 0.05 level) on regression slopes, regression offsets, and *R*^2^ values (Data S5 and S6, supplemental information). The calculations suggest that angular changes of most leg joints are broadly similar in all strains (for exceptions see Data S6, supplemental information). We found most of the significant changes for the femur tibia angle δ with significant differences for regression slope and offset (Figure 6). The initial femur tibia angle at zero load (regression offset) varies between *act5C-*cas9 flies and the strains *wild type*, *R104*, and *res*^*+*^*-*Venus, respectively. The same holds for *res*^*–*^-knockout strains and the latter. Significant differences were also obtained for the rate of angular change (regression slope) between *R104* controls and *wild-type*, *act5C-*cas9, *res*^*+*^*-*Venus, and *res*^*–*^ strains, respectively. Altogether, the data appear somewhat mixed and suggest only subtle changes in leg folding behavior in both the control and resilin-modified strains. The most significant finding is that both *res*^*+*^*-*Venus and *res*^*–*^ strains significantly differ from each other and both lines are different from the *R104* control strain (*p* < 0.05). Noteworthy, although all leg angles change to some degree, they, in part, compensate each other because both the initial effective leg length and the rate of change during compression is not significantly different among the strains, except for the pairing *wild-type* vs. *res*^*+*^*-*GFP flies (Data S6, supplemental information). In conclusion, the latter findings predict only subtle differences in body posture in sitting animals in all tested strains.

### Work-loop analysis, leg stiffness, and elasticity

**Figure 7:**
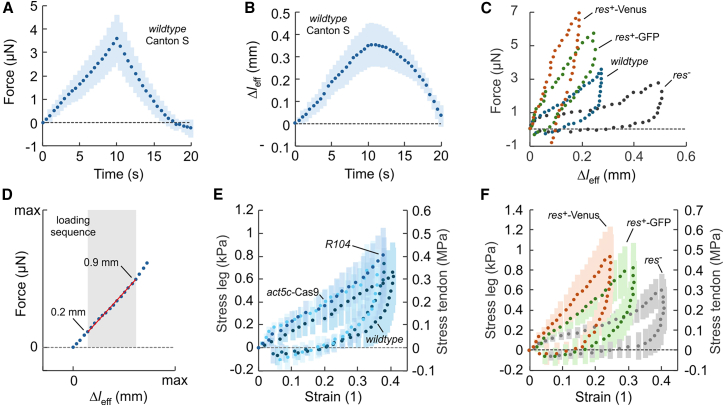
Stiffness and elasticity measurements of the *Drosophila* middle leg (A) Typical time trace of leg force development within the 20 s measurement time of 11 *wild-type* Canton S flies. Leg compression (0*–*10 s) and relaxation (10*–*20 s) occurs in 40 steps of 0.05 mm body displacement. (B) Corresponding changes in effective leg length for data shown in (A). (C) Typical work-loops of force development and change in effective leg length (Δ*l*_eff_) of single animals and four strains. Note the different slopes during loading and unloading sequences. (D) Total leg stiffness and elasticity are calculated from data within a linear range (gray, 0.2–0.9 mm) of the work-loop during force loading. (E) Comparison of stress-strain work-loops for the three control strains. Stress is calculated by dividing the mechanical force by mean outer diameter of the middle leg including femur, tibia, and tarsi (left scale) or mean diameter of the long tendon (right scale, see Data S3, supplemental information). (F) Work-loops with averaged data for resilin overexpression strains *res*^*+*^*-*Venus (red) and *res*^*+*^*-*GFP (green) and for the *pro-resilin*-knockout strain *res*^*–*^ (gray). See (E) for explanations of scales. Statistics is shown in Data S7–S8, supplemental information. Data are represented as mean ± standard deviation (colored areas in A, B, E, and F). *n* = 11 *wild type*, *n* = 7 *R104*, *n* = 9 *act5C*-cas9, *n* = 12 *res*^*+*^-GFP, *n* = 11 *res*^*+*^-Venus, and *n* = 15 *res*^*-*^ flies.

**Figure 8:**
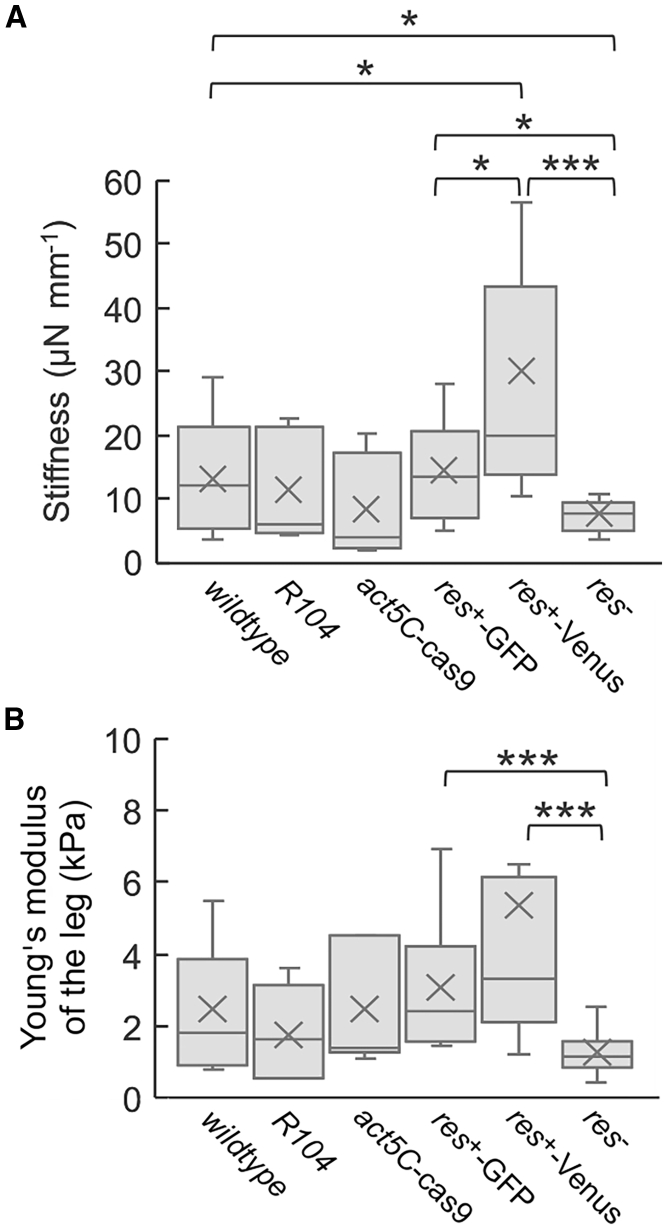
Stiffness and Young's modulus of the *Drosophila* middle leg Boxplots of (A) mean leg stiffness and (B) leg elasticity from work-loop analysis in the 6 tested strains of fruit flies Data are represented as boxplot showing interquartile range (25th–75th percentiles) with whiskers extending to 5th and 95th percentiles. Medians, horizontal line; means, cross. Data were tested for normality using a Shapiro-Wilk test and depending on the test outcome, data differences either by Mann-Whitney U test or *t* test (see also Data S8, supplemental information). ∗∗∗*p* < 0.001, ∗*p* < 0.05. *n* = 11 *wild type*, *n* = 7 *R104*, *n* = 9 *act5C*-cas9, *n* = 12 *res*^*+*^-GFP, *n* = 11 *res*^*+*^-Venus, and *n* = 15 *res*^*-*^ flies.

Besides leg folding, the experimental setup in Figures 1A–1C allowed us to measure both leg displacement and leg loading force, and from the ratio between both measures, we calculated leg stiffness. We modeled the leg as a compressible suspension strut connecting the coxa with the pretarsus (Figure 1B, see “method details” section for more details on the experimental setup). We found that although leg angles vary only little among strains during force loading, the overall stiffness of the fly leg significantly changes depending on the tendon’s resilin content. Figure 7A shows a typical time trace of leg force within a force loading (0–10 s) and unloading (10–20 s) cycle in *wild-type* animals. Figure 7B shows the corresponding change in effective leg length. We found that in all fly strains, the loading slope is more linear than the unloading slope, suggesting differences in stiffness property during leg compression and relaxation. From the linear range between 0.2 mm and 0.9 mm leg displacement during force loading (compression), we estimated leg stiffness and elastic modulus (Young’s modulus). The slope during leg force unloading is typically divided into two phases with an elevated decrease in force during the early phase of relaxation at Δ*l*_eff_ = 1.0–0.75 mm and a slight decrease below Δ*l*_eff_ = 0.75 mm (Figures 7C–7F). These differences during loading and unloading may even produce slight negative forces during relaxation below Δ*l*_eff_ = 0.25 mm (Figure 7C), at which the leg is slightly pulling the sensor wire. Work-loops of a full leg compression-relaxation cycle in single flies are shown in Figure 7C. Unlike leg angles, the data show tremendous differences in force development. We found minimum stiffness (maximum compliance) in the knockout strain *res*^*–*^ (7.75 ± 3.78 μN mm^−1^; Data S7, supplemental information) that is ∼3.9-fold less than maximum leg stiffness in *res*^*+*^*-*Venus (30.1 ± 23.2 μN mm^-1^) during leg compression (Figure 7F). *Wild-type* flies (13.1 ± 7.97 μN mm^-1^) and *res*^*+*^*-*GFP strains (14.3 ± 7.94 μN mm^−1^; Data S7, supplemental information) lie between these two extremes (Figures 7C, 7E, and 8A). Statistical testing suggests significant differences between (1) *wild-type* controls and resilin overexpression strains (*res*^*+*^*-*Venus), (2) *wild-type* and knockout strains (*res*^*–*^), (3) overexpression and knockout strains, and (4) the two resilin overexpression strains (*res*^*+*^*-*Venus and *res*^*+*^*-*GFP; Data S8, supplemental information). Moreover, all control strains are similar (Figures 7E; Data S9, supplemental information). The above values convert into a Young’s modulus using strain normalized to either the mean diameter of (1) the leg (left scale, Figure 7F) or (2) the long tendon (right scale, Figure 7F). Young’s modulus varies between 1.29 ± 0.62 kPa and 0.69 ± 0.33 MPa, respectively, in *res*^*–*^ flies and 5.35 ± 5.36 kPa and 2.85 ± 2.85 MPa, respectively, in *res*^*+*^*-*Venus flies (Figure 8; Data S7 and S9, supplemental information).

The increase in Young’s modulus with increasing *pro-resilin* expression is unexpected because superficially an overexpression of *pro-resilin* should make a leg more compliant and not stiffer. In insects, cuticle stiffness varies between 1 kPa in hydrated intersegmental membranes of locust,^27^ ∼1.0 MPa in hydrated resilin,^38^ ∼60 MPa in the abdominal cuticle of *Rhodnius*,^39^ and more than 10 GPa in the well-tanned isotropic tendon of locust.^40^^,^^41^ A Young’s modulus of 2.85 MPa for axial stress (tendon stress; Data S7, supplemental information) is thus at the low end of this range. However, an elevated number of crosslinks due to a higher content of an elastic protein could enhance Young’s modulus. This has been demonstrated in collagen films, in which the crosslinked protein yields a ∼5-fold higher stiffness than the non-linked protein.^42^ Other evidence includes the elevated concentration of elastin in the mesentery of embryos that also increases tissue stiffness.^43^ Altogether, the expression strength of *pro-resilin* inside the leg tendons of *wild-type* flies might reflect the best compromise between leg stiffness and compliance for motor behavior and body posture control, and might thus yield the best locomotor efficiency during jumping and walking. Collectively, the suggested role for resilin in the long tendon might be 2-fold: it allows tendon elongation at the joints during flexion of the tarsi and helps to shorten the tendon in order to support the stretching of the tarsi when the tendon muscles relax.^4^^,^^18^^,^^44^^,^^45^ A change in resilin content could somewhat alter this elastic recovery, which might explain the different stiffness behaviors of the leg during a force loading-unloading cycle in the biomechanical experiments.

Noteworthy, resilin is not the only elastic protein of the chitin matrix that potentially contributes to the mechanical properties of the leg but there is also cpr56f, as mentioned in the introductory section. Unlike *pro-resilin*, *cpr56f* is expressed before metamorphosis, and thus mutations in the *cpr56f* gene are probably lethal at the larval stage, precluding analyses of mutant adults. Our previous work using qPCR, moreover, suggests a complex interaction between the expression patterns of both genes.^17^ In flies, in which the levels of *cpr56f* transcript are reduced by RNAi, the expression of *resilin* is slightly upregulated, suggesting a compensatory mechanism. By contrast, in flies in which the levels of *resilin* transcript are reduced by RNAi, the expression of *cpr56f* is also reduced. Despite the mentioned restriction, a double knockdown experiment could here serve as a test bed to better understand the combinatorial functions of *resilin* and *cpr56f* for leg function in the fruit fly.

In a last step, we estimated the leg’s work on the sensor wire during force loading (compression, negative work) and unloading (relaxation, positive work) that allowed us to estimate the loss of elastic potential energy during a full leg folding-unfolding cycle. These data are summarized in Data S8, supplemental information, and suggest that negative work ranges from −0.47 ± 0.15 nJ in *Act5C-cas9* to −0.71 ± 0.20 nJ in *wild-type* controls. Positive work during leg expansion is rather similar in all tested strains despite the different work-loop shapes, with a maximum value of 0.21 ± 0.13 nJ in *wild-type* controls. Most noteworthy, negative and positive work was almost identical in *res*^*+*^*-*Venus and *res*^*–*^ strains (Data S8, supplemental information). We found, moreover, no significant differences in the loss of elastic potential energy among resilin-modified and control strains because all values are scattered around an elevated relative loss of approximately −72% (Datas S8 and S9, supplemental information for statistics). This means that even though leg stiffness varies severalfold depending on resilin content, all legs superficially seem to suffer from the same tremendous loss in elastic potential energy under the given experimental condition. This loss of elasticity of the leg is more than ∼3.5-fold higher than the elasticity loss in resilin-containing fly wings under similar experimental conditions (*Calliphora*, ∼20%^46^). Possible explanations for our finding include not only plastic deformation of tendon and cuticle but also changes in active control of leg stiffness via the FCO. The receptor neurons of FCO are activated by motion of the moveable arculum via two sensory ligaments inside the femur (Figure 4F). The arculum provides feedback for the angular position of the coxo-femoral and femoro-tibial joints. Stretching the femoro-tibial joint results in rotation of the arculum and a sequential activation of FCO receptor cells in *Drosophila*.^37^ A previous study has shown that the inhibition of FCO’s club and hook neurons alters leg kinematics and phase during walking.^47^ Similarly, genetic ablation of the FCO in *Drosophila* (*nanchung*^48^) results in defects of gait and leg control. We thus hypothesize that the non-linear leg stiffness during force unloading and also the subtle negative leg force at the end of the sequence (Figure 7) might not reflect the properties of leg tendons and muscles but the changes in control of leg muscle tension by FCO. As resilin locally resides in the arculum tendon (Figure 4F), changes in resilin concentration could even alter the set point of the leg posture control loop and thus leg stiffness, which might eventually hamper jumping and walking behavior in the fruit fly. It is less likely that muscle properties change in our transgene animals because the development of embryonic muscle cells is largely finished (mid-embryogenesis) when obstructor chitin-binding proteins, chitin deacetylases, chitinases, and matrix-protecting proteins such as knickkopf (knk) and retroactive (rtv) are formed (late embryogenesis).^23^^,^^27^^,^^31^

In conclusion, the goal of this study was to demonstrate the impact of elastic resilin on the biomechanics of leg motion in *Drosophila*. Our data suggest that resilin inside the leg of *Drosophila* resides in the chitin/chitosan-matrix of several muscle tendons and is not located in the outer cuticle as predicted before.^17^ The combined behavioral-genetics approach implies that a significant overexpression of *pro-resilin* increases total leg stiffness approximately 4-fold compared to *pro-resilin*-knockout animals, while leg elasticity remains unaffected and similar to *wild-type* flies. Despite this vast change in stiffness, leg folding angles vary little between strains suggesting little change in leg posture of sitting flies. However, the increase in leg stiffness is counterintuitive, as elastic proteins are thought to make the locomotor system more compliant, allowing larger changes in tendon lengths at lower forces without plastic deformation.^3^ However, to link *pro-resilin* expression to leg function in the context of a living and behaving system rather than to test material properties eventually requires more research. This could include experiments in flies with ablated FCO receptor cells and behavioral tests in *resilin-cpr56f* double-knockout flies, including experiments on running capacity and the metabolic costs of running. Eventually, it remains unclear which structural components are responsible for the resilin-mediated changes in our biomechanical assay, as resilin is found in several tendons of the leg. The exact function of resilin within the locomotor apparatus of the insect thus remains controversial, in particular due to the complex interaction between mechanical structures for leg motion and their sensorimotor control.

### Limitations of the study

Biomechanical measurements are conducted with semi-intact animals. Leg motion and control might thus differ in a freely behaving animal. We also only tested middle legs of the animal because these legs experience maximum forces of up to 81-fold the body weight during take-off behavior for flight. As the morphology of fore- and hindlegs are somewhat different, we cannot exclude differences in stiffness and elasticity compared to middle legs. Moreover, our data apply to female flies, and our findings should thus be confirmed in males.

## Resource availability

### Lead contact

Further information and requests for resources and methods should be directed to the lead contact Fritz-Olaf Lehmann (fritz.lehmann@uni-rostock.de).

### Materials availability

Animals are available from the lead contact upon request.

### Data and code availability

The authors declare that the data supporting the findings of this study are available within the article.

• All data reported in this paper will be shared by the lead contact upon request.
• This study does not report original code.
• Any additional information required to understand the results are available from the lead contact upon request.

## Acknowledgments

We thank Bärbel Redlich-Witt for her help with fly breeding. This work was financially supported by grants LE905/19-1 to F.O.L. and MO1714/15 to B.M. from the Priority Program “Code-Chi – Chitin, chitosan and chito-oligosaccharides and their interaction with proteins of the extracellular matrix and cellular signaling” (SPP2416) of the German Research Foundation (DFG).

## Author contributions

Conceptualization, S.O. and F.-O.L; methodology, S.O.; formal analysis, S.O.; investigation, S.O.; resources, B.M. and F.-O.L.; data curation, S.O. and F.-O.L.; writing – original draft, S.O.; writing – review & editing, B.M. and F.-O.L.; visualization, S.O.; project administration, F.-O.L.; funding acquisition, B.M. and F.-O.L.

## Declaration of interests

The authors declare no competing interests.

## STAR★Methods

### Key resources table

**Table:** 

REAGENT or RESOURCE	SOURCE	IDENTIFIER
**Chemicals**		

Cyanacrylate	Henkel	https://www.pattex.de
Direct Red 23	Sigma-Aldrich	http://www.sigmaaldrich.com
PBS	Sigma-Aldrich	http://www.sigmaaldrich.com

**Experimental models**		

*Drosophila melanogaster* Canton S	F.O.L.	N/A
*D. melanogaster* curly *R104*	stock: *Bl,*^1^*L*^*2*^/*CyO*	https://bdsc.indiana.edu/index.html
*D. melanogaster act5C*-cas9	Bloomington #58492	https://bdsc.indiana.edu/index.html
*D. melanogaster res*^*+*^-GFP	B.M.	see STAR Methods section
*D. melanogaster res*^*+*^-Venus	B.M.	see STAR Methods section
*D. melanogaster res*^–^	B.M.	see STAR Methods section

**Software and algorithms**		

ImageJ	ImageJ	https://imagej.net/ij/
MATLAB® 2018b	The Mathworks	https://mathworks.com
Octave V4.0.0	GNU	https://ftpmirror.gnu.org/octave
CoreDRAW X6	Corel Corporation	https://www.coreldraw.com
Origin 8.5.1	OriginLab	https://www.originlab.com/

### Experimental model and study participant details

All experiments were conducted with virgin 3 to 5-day-old female *Drosophila melanogaster*. The animals stem from strains in our laboratory and were bred on instant cornmeal (Formula 4-24, Burlington, Carolina, USA) at 24°C ambient temperature, 80% humidity and a 12/12 h day/night cycle. *Wild-type* animals are long-term bred strains, while transgene strains as listed in the key resources table were provided by B.M. In total, we scored 65 flies on resilin-dependent leg stiffness and 40 flies on the distribution of resilin in the long tendon and fluorescence of the cuticle.

We tested several genetically modified fly strains with different expression rates of *pro-resilin* due to various copies of the *pro-resilin* gene (*Res,* CG15920): (i) two strains with multiple copies of the *pro-resilin* gene, (ii) a *pro-resilin* knockout strain, (iii) parent lines of the latter and (iv) *wild type* flies. The genetics of the strains has previously been published and thus we only provide a short description.^17^ For *pro-resilin* overexpression, we generated flies with a transposon plasmid (PBac) containing the *pro-resilin* (41.9 kb) genomic DNA with a green fluorescent protein (GFP) coding region at position 43A1 on the left arm of chromosome 2. In a second approach, we cloned the *pro-resilin* coding region into a pTWV transposon plasmid seamlessly upstream of the sequence coding for Venus fluorescent protein. The UAS-*pro-resilin-*Venus construct was inserted into *w*^*1118*^ and the animals crossed with daughterless *da*-Gal4 promoter strains for ubiquitous protein overexpression (*res*^*+*^*-*Venus strain). The curly balancer *CyO* (stock: *Bl*,^1^ *L*^2^/*CyO*) was used to genetically stabilize the strains. *Pro-resilin* knockout (*res*^*–*^ strain) was generated by gene editing applying the Crispr/Cas9 method. A gDNA (g4) directed against the ATG of the gene was injected into act-*cas9* embryos (Bloomington stock #58492). Single male flies with held-down wings, as observed in RNA interference flies with reduced *pro-resilin* expression, were collected to establish stable stocks. By sequencing of genomic DNA, a missense mutation in *pro-resilin* close to the ATG was identified.^17^ For this, we cloned g4 DNA into a pCFD3-dU6/3 vector and injected this construct into cas9-expressing embryos. For comparison, we tested the genetic parent lines with the actin 5 promotor (*act5C-*cas9) and curly *R104,* and *wild*-*type* Canton S as controls. For the reported experiments, no approval of the local institutional ethics board (ethic board of Rostock University medicine) was required.

### Method details

#### Tendon preparation and staining

For analysis, we removed the long tendon from the mesothoracic leg by holding the distal tarsal segments with a tweezers and gently pulling the tendon out of the leg (Data S1, supplemental information). To avoid desiccation, the tendons were immediately transferred into a water bath and subsequently mounted on a glass plate for microscopic imaging. While pulling, the long tendon separates from its attachment sites of the long tendon muscles 1 and 2 inside the femur and tibia, respectively. Identification of tendon muscles occurred according to a previously published study on *Drosophila* leg muscles.^27^ In genetically modified lines, the tendon often disrupted during pulling at the transition (joint) between tibia and 1^st^ tarsus, which made it difficult to exactly estimate tendon length and structure. We found that in *wild-type* flies, the isolated tendon is 8% shorter than the leg (tendon, 1.70 ± 0.07 mm; corresponding leg length, 1.85 ± 0.02 mm, *n* = 10 flies; *t-*test, *p* = 0.023) suggesting no lengthening of the tendon owing to the preparation. Instead, the data suggests that the tendon is under tension in the stretched leg.

As resilin in insects typically occurs as a composite material in the chitin-matrix, we stained in several *wild-type* flies the long tendon with Direct Red 23, in order to prove chitin as the main tendon substrate.^49^ The procedure was as follows: the tendon was removed from the leg, transferred into 0.01% dye solution for 4 min, washed with water and phosphate-buffered saline and eventually embedded in glycerol for microscopy. Presence of chitin in the tendon was also confirmed by staining with the chitin dye Congo Red (data not shown).

#### Microscopic imaging

For imaging, we used different microscopes. To validate resilin autofluorescence, we used a fluorescence microscope (Axio Imager Z1, Zeiss) equipped with a DAPI filterset (365 nm peak excitation, 445/50 nm emission filter, 395 nm FT beam splitter) and Zen 2.3 software (Figure 5). The structure of the tendon was investigated with a bright-field phase-contrast microscope (Axioskop 2 F S plus, Zeiss) equipped with a 100× dry objective (Figures 3 and 5). This microscope was also used to generate combined bright-field and epi-fluorescence (HBO100 mercury lamp; Zeiss filterset 38, excitation 470/40 nm, beamsplitter FT 495 nm, emission 525/50 nm) images of structure and resilin distribution in *res*^*+*^*-*GFP strains. Photos were taken by a single-lens reflex camera (EOS 750D, Canon) mounted to the microscope at a resolution of 6000 × 4000 image pixels. For imaging chitin autofluorescence together with GFP fluorescence, we employed a confocal microscope (TCS SP8, Leica) with a 488 nm fixed wavelength laser (OPSL 488, Leica) for excitation and 566–695 nm and 500–550 nm emission windows for chitin autofluorescence and GFP, respectively (Figure 4). For Direct Red 23 imaging, excitation was also at 488 nm but the emission window was 590–600 nm. Confocal images were captured at a resolution of 2048 × 2048 image pixels. We verified expression rates of *pro-resilin* from fluorescence of the tyrosine bonds in the synthesized resilin.^17^ It has been suggested that 20%–25% of pro-resilin molecules are crosslinked by tyrosine bonds.^11^^,^^35^

#### Stiffness and elasticity measurements

For measurements of leg stiffness and elasticity, we developed a biomechanical assay for testing semi-intact living animals (Figure 1). We selected the mesothoracic leg for testing because this leg produces elevated forces during take-off jumping of up to 137 μN compared to pro- and metathoracic legs.^50^^,^^51^^,^^52^ During landing, each leg of fruit flies experiences up to 47 μN load, and the estimated leg loading in a sitting fly is 1.7 μN at 10 μN body weight.^53^ To maintain its neural motor control and sensory pathways, the tested leg was not removed from the fly body but remained attached. This approach, moreover, uses the leg’s natural attachment site on the body and avoids desiccation of the leg and its internal structures. As the fly was alive during the tests, however, the stiffness measurements were often disrupted by voluntary leg movements for running or grooming. To avoid these movements, we removed the head of the fly that interrupts the transfer of locomotor commands generated by the central nervous system.^54^^,^^55^ The entire dissecting procedure was as follows: we anesthetized the intact animal on crushed ice for 5 min and removed the head including all legs near the coxa, except for the left mesothoracic leg. Wounds were quickly sealed with superglue (Pattex superclue, Henkel). The entire fly body was then glued to a 0.4 mm diameter holder with superglue and incubated for approximately 10–15 min in a container with elevated humidity before testing the fly in the setup. The bodies were also incubated in the container between multiple experimental runs.

Figure 1A shows a sketch of the experimental setup. The holder with the animal is attached to a computer-controlled piezo-driven micromanipulator (micro positioning table M-122.2DD1, mercury step C-663, Physik Instrumente, Germany) that allows to move the fly toward a force sensor. The sensor is a 40 mm long (*e*, Figure 1C) and 50.8 μm diameter tungsten wire with a free end (single point load cantilever). Wire bending under tip load was calibrated prior the experiment with small weights ranging from 0.8 mg to 3.54 mg (linear regression fit, *R*^2^ = 0.99, *p* < 0.001; Methods S10, supplemental information). Maximum wire deflection was 0.81 mm at 5.37 μN force applied to a fly leg. At start of a measurement, the sensor was manually positioned in *xyz*-direction by a micro-manipulator until the 5^th^ tarsus of the unloaded leg made contact to the wire (Figure 1C). A small crossbar mounted normal to the sensor wire at the leg’s attachment point hindered the leg to move sideways but otherwise the leg was not mechanically restrained and not glued to the wire. Noteworthy, the initial angles between leg segments varied somewhat in unloaded legs of each strain (linear regression offset; Data S5, supplemental information). Besides the different contents of resilin, we attribute part of this to the loss neural feedback from leg muscle strain receptors lacking gravitational loading by body mass.^37^^,^^56^ The horizontal distance between leg-wire contact point and coxa was not significantly different among the tested lines (length *d*, Figure 1C; unpaired *t-*test, *p* > 0.05; Data S3, supplemental information).

We recorded leg folding and wire deflection with a video camera (acA720-290 gm, Basler) mounted to a dissecting microscope (Stemi 508, Zeiss) with its optical axis orientated normal to the leg folding plane. Recording frame rate was 10 Hz and image resolution 720 × 540 video pixels. After the initial leg-wire contact, we moved the piezo-driven micromanipulator 1.0 mm toward the sensor wire in steps of 0.05 mm to increase stress on the leg (leg compression). Due to wire bending, however, effective leg compression step width (*b*, Figure 1C) varied somewhat depending on leg stiffness. After each step, the movement stopped for 0.5 s allowing recording of 5 video frames at constant load. After a complete loading sequence, we reversed the direction of motion and recorded leg position while unloading. A complete loading-unloading cycle was 20 s. As pre-tests with repetitive cycling showed no difference between individual runs, we measured only one complete cycle in each animal. From the video images, we reconstructed the leg’s folding behavior by automatically tracking major tarsal and coxa markers and manually remaining leg joints using a video tracking software based on image matching (Tracker V6.3.2, Figures 1B and 1C). Leg position for each compression step is tracked in 5 subsequent video frames to minimize tracking errors (intraclass correlation coefficient = 0.99).^57^^,^^58^ Control experiments with long-lasting constant 2.23 μN loading force suggest no significant fatigue of leg stiffness during leg compression over 20 s measurement time (571 loading cycles, *n* = 16 *wild-type*, *n* = 15 *res*^*+*^*-*GFP, *n* = 12 *res*^*+*^*-*Venus, and *n* = 11 *res*^*–*^). Leg lengths, leg extension and leg extension angles of all tested lines are shown in Data S3, supplemental information. We found no differences in the latter parameters (i) among resilin-modified strains and (ii) between these animals and their genetic parent strains, and only small differences between *wild*-*type* flies and resilin-overexpression strains (unpaired *t-*test, *p* = 0.03–0.04; Data S3, supplemental information).

#### Work-loop analysis

For quantification of the leg’s biomechanical properties, we modeled the leg as a compressible suspension strut connecting the coxa with the pretarsus (Figure 1B).^59^ The properties of all leg joints (bending stress) and leg segments (axial stress) are thus lumped together into a single value. We have chosen this approach because it allows us to characterize the behavior of the entire leg instead of a single joint. The approach is restricted, however, in a way that it does not allow measurements of rotational stiffness of leg joints but only provides an estimate of total spring stiffness in the direction of force loading. We did not attempt to correct leg position prior the measurement or to move the tarsal end to the desired position on the tungsten wire. Instead, we started the measurements with leg joint angles as they appeared in the decapitated fly when the legs had no ground contact and were unloaded. As initial joint angles are not significantly different among the strains, except for angle *δ* (femoro-tibial joint; Data S6, supplemental information), and the initial leg extension angle α is not correlated with maximum leg force (Data S4, supplemental information), the differences in joint-specific contribution to total leg stiffness are thought to be small among the tested strains. Assuming this simplification, we defined the leg’s effective stance force and length change (strain) as follows. Effective leg length (*l*_eff_) and thus leg shortening was calculated from distance between coxa and sensor wire (*b*, Figure 1C) and leg extension angle α (*l*_eff_ = *b*/cos(α); Figure 1C). Leg force consists of components normal (*F*_n_) and parallel (*F*_p_) to the wire, whereby the leg could not slip sideways (see above, Figure 1C). The normal component may deflect the sensor wire and effective force (*F*_eff_) was thus calculated from the ratio between *F*_n_ and cos(α). At maximum wire deflection, a cantilever numerical model predicts a wire slope at the leg’s contact point of less than −1.7°, which was ignored in subsequent calculations.

For estimations of stiffness (elastic modulus) and elasticity (loss of elastic potential energy) of the leg, we adopted the work-loop approach developed for studies on muscle force production in insects.^60^ For estimation of Young’s modulus, we transformed *F*_eff_ into stress by dividing *F*_eff_ through either the leg’s or the long tendon’s mean cross-sectional area (CSA). We estimated CSA from bright-field images using the *general image fiber tool macro* (GIFT) for Fiji image processing software (https://fiji.sc/).^61^^,^^62^ We also assumed that leg and tendon are circular. Mean leg and tendon diameters are the peak of a Gaussian model curve fitted to the histogram of all local diameters of femur/tibia/tarsi and the complete long tendon, respectively. Eventually, following Hooke’s law, the material’s elastic or Young’s modulus is equal to the ratio between stress and strain in the linear data range. A loss of elastic potential energy Δ*E*_pot_ of the leg results from changes in stiffness during the loading-unloading cycle. Δ*E*_pot_ is calculated from the total difference in absorbed work during force loading (negative work) and the work released during unloading (positive work). These values are expressed by the areas under the loading-unloading curve in the force-length diagram.

### Quantification and statistical analysis

All calculations and data analyses were done in Excel (16.99.1, Microsoft, USA), Origin (8.5.1, OriginLab, USA) and by self-developed software routines written in MATLAB (R2024a, The MathWorks Inc, USA) and Octave (GNU, GPL 3). For statistics, we employed Shapiro-Wilk tests for testing on normality and two-side unpaired *t*-tests (normality) or Mann-Whitney-U tests (no normality) for data comparisons. Differences between multiple means are tested by ANOVA and significances by Tukey post-hoc test. Significance was tested at the levels *p* < 0.001 (∗∗∗), *p* < 0.01 (∗∗) and *p* < 0.05 (∗). If not stated otherwise, data are shown as means ± standard deviations.
