# Supplementary material for: Levels of elastic resilin modulate leg stiffness but not elasticity in Drosophila
Source: iScience. 2026 Jun 17;29(7):116404. doi: 10.1016/j.isci.2026.116404 (PMC13310938; doi:10.1016/j.isci.2026.116404)
Supplement: Document S1. Figures S1 and S10, Tables S2–S9, Data S1–S9, and Methods S10 [file mmc1.pdf]

**iScience, Volume *29***

# Supplemental information

# Levels of elastic resilin modulate leg stiffness but not elasticity in *Drosophila*

**Sarah Oeftger, Bernard Moussian, and Fritz-Olaf Lehmann**

### Data S1: Fluorescence of leg with and without long leg tendon

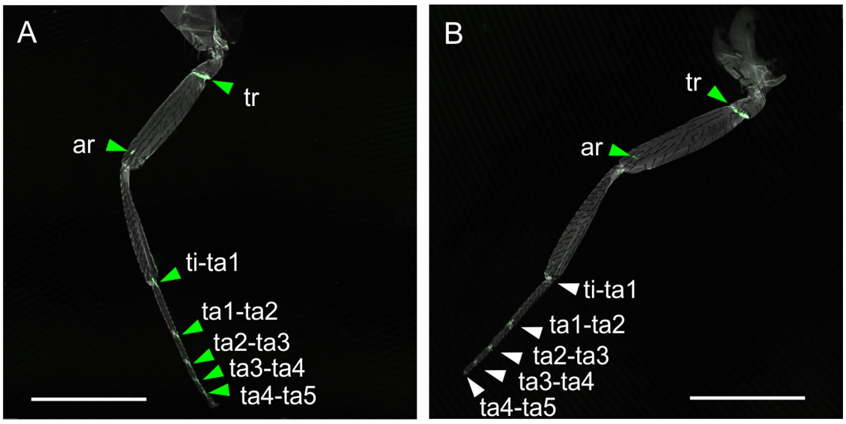


**Figure S1.** GFP-fluorescence of *pro-resilin* in a *Drosophila* middle leg expressed in a *res*$^{+}$-GFP animal. (A) The intact leg shows GFP-fluorescence at the tarsal joints, at the arculum and the trochanter. (B) After removal of the long leg tendon, there is no fluorescence at the tarsi, while fluorescence of the tibia-tendon at the arculum and tendons of the trochanter remains. White and green arrows indicate cuticle autofluorescence and GFP-signaling, respectively. Both images are captures by a multi-photon microscope with same settings for autofluorescence and GFP-signaling (*cf.* methods details of the main text). Scale bars are 600 µm. *Related to figures 3 – 5.*

### Data S2: Validation of genetics

| *Fly strain* | ROI 1 (0 - 255) | ROI 2 (0 - 255) | ROI difference (0 - 255) |
|---|---|---|---|
| *Wildtype* | 46.8 ± 10.9 | 24.0 ± 11.1 | 22.8 ± 7.53 |
| *res*$^{+}$-GFP | 53.4 ± 10.4 | 20.5 ± 4.42 | 32.9 ± 8.95 |
| *res*$^{+}$-Venus | 60.3 ± 9.69 | 25.7 ± 7.08 | 34.6 ± 6.56 |
| *res*$^{-}$ | 7.34 ± 2.54 | 0.32 ± 0.52 | 7.02 ± 2.57 |

**Table S2.** Combined autofluorescence of cuticle and resilin using a DAPI filterset (365nm peak excitation, 445/50 nm emission filter, 395nm FT beam splitter) in two regions-of-interest (ROI) of the fly leg (*cf.* figure 2 of the main text). Fluorescence is shown in absolute units (0 [dark] - 225 [bright]). ANOVA is used for statistical comparison. The ROI differences of *wildtype*, *res*$^{+}$-GFP and *res*$^{+}$-Venus do not significantly differ from each other, while the knockout strain *res*$^{-}$ is significantly different from the remaining strains. ROI 1, cuticle at the trochanter (red, figure 2); ROI 2, background fluorescence at femur (green, figure 2); N = 10 animals of each strain. Data are represented as mean ± standard deviation. *Related to figure 2.*

### Data S3: Leg morphology and posture during force loading

| *Fly strain* | Leg length (mm) | Mean leg diameter (µm) | Leg extension *d* (mm) | $l_{eff}$ at 0 µN | *α* (degree) | N |
|---|---|---|---|---|---|---|
| *wildtype* | 2.03 ± 0.02 | 75.1 ± 4.19 | 0.46 ± 0.17 | 0.94 ± 0.13 | 14.9 ± 7.84 | 11 |
| *R104* | 2.05 ± 0.03 | n.e. | 0.54 ± 0.17 | 0.93 ± 0.25 | 20.6 ± 6.95 | 9 |
| *act5C*-cas9 | 2.05 ± 0.02 | n.e | 0.70 ± 0.43 | 1.10 ± 0.14 | 24.3 ± 12.8 | 7 |
| *Res*$^{+}$-GFP | 2.05 ± 0.03 | 76.7 ± 5.37 | 0.65 ± 0.22 | 1.00 ± 0.23 | 22.9 ± 8.57 | 11 |
| *res*$^{+}$-Venus | 2.04 ± 0.03 | 74.2 ± 3.22 | 0.63 ± 0.18 | 0.96 ± 0.20 | 21.9 ± 6.94 | 12 |
| *res*$^{-}$ | 2.03 ± 0.03 | 76.1 ± 2.85 | 0.50 ± 0.16 | 0.98 ± 0.22 | 17.9 ± 8.28 | 15 |

**Table S3**. Leg morphology and posture during biomechanical loading measurements. Leg length, sum of all segment lengths from mid trochanter to tip of 5$^{th}$ tarsus; leg extension, horizontal distance (*d*, Fig. 1C) between coxa and leg-wire contact position; $l_{eff}$, effective leg length (Fig. 1C); *α*, leg extension angle (Fig. 1C). None of the table data are significantly different among fly lines (*t*-test, $p > 0.05$) except for *wildtype* Canton S vs. *act5C*-cas9 (*t*-test; *d*, $p = 0.04$; *α*, $p = 0.03$) and *wildtype* vs. *res*$^{+}$-GFP (*t*-test; *d*, $p = 0.03$; *α*, $p = 0.03$). Mean leg diameter was estimated from the outer cuticle diameter of femur, tibia and tarsi in 10 flies each. n.e., not estimated; means ± standard deviation. Data are represented as mean ± standard deviation. *Related to figure 3.*

## Data S4: Linear regression statistics between leg extension angle and leg force

| *Fly strain* | Slope (μN $deg^{-1}$) | Offset (μN) | Pearson *r* | *F*-value | Probability *p* | N |
|---|---|---|---|---|---|---|
| *Wildtype* | 0.024 ± 0.031 | 2.91 ± 0.94 | 0.26 | 0.58 | 0.47 | 11 |
| $res^+$-GFP | 0.043 ± 0.023 | 2.15 ± 0.82 | 0.54 | 3.69 | 0.09 | 11 |
| $res^+$-Venus | 0.030 ± 0.042 | 3.28 ± 1.36 | 0.22 | 0.50 | 0.50 | 12 |
| $res^-$ | 0.006 ± 0.012 | 2.44 ± 0.43 | 0.14 | 0.24 | 0.63 | 15 |

**Table S4**. Linear regression statistics between the initial leg extension angle *α* (*x*-scale) when no force is exerted on the sensor wire and maximum leg force (*y*-scale) after displacing the leg by 1.0 mm (*cf.* Fig. 6 of main text). If maximum force depends on extension angle, the slope of the linear regression line would be unequal to zero. A *p*-value larger than 0.05 indicates that the linear model (slope ≠ 0) is not statistically significant. *F*-value, test value of the ANOVA *F*-test; *p*, probability that the slope explains a significant amount of variance compared to a flat regression line; N, tested flies of each strain. Data are represented as mean ± standard deviation (slope, offset) and statistics to means (Pearson, *F*-value, probability). *Related to figures 1 and 6.*

## Data S5: Linear regression fit statistics on leg angles and effective leg length

| *Strain* | Parameter | Mean slope (degrees $μN^{-1}$), (μm $μN^{-1}$) | Mean offset (degrees), (μm) | Mean $R^2$ |
|---|---|---|---|---|
| *Wildtype* | $γ$ | 5.99 ± 2.72 | 17.0 ± 10.2 | 0.94 ± 0.04 |
| *N* = 10 flies | $δ$ | -4.84 ± 1.68 | 46.2 ± 7.90 | 0.86 ± 0.15 |
| | $ε$ | -0.31 ± 5.22 | 161 ± 9.08 | 0.67 ± 0.17 |
| | $η$ | 1.05 ± 3.37 | 85.2 ± 7.54 | 0.72 ± 0.29 |
| | $l_{eff}$ | -101 ± 55.0 | 950 ± 146 | 0.93 ± 0.09 |
| *R104* | $γ$ | 4.61 ± 3.11 | 21.6 ± 13.6 | 0.86 ± 0.07 |
| *N* = 9 flies | $δ$ | 0.19 ± 3.39 | 43.4 ± 7.69 | 0.60 ± 0.31 |
| | $ε$ | -3.65 ± 6.69 | 169 ± 15.5 | 0.68 ± 0.27 |
| | $η$ | -1.62 ± 3.06 | 84.1 ± 11.1 | 0.83 ± 0.16 |
| | $l_{eff}$ | -57.1 ± 37.3 | 915 ± 250 | 0.89 ± 0.06 |
| *act5C*-cas9 | $γ$ | 1.81 ± 3.41 | 10.7 ± 11.6 | 0.80 ± 0.20 |
| *N* = 7 flies | $δ$ | -5.33 ± 3.09 | 69.9 ± 7.87 | 0.86 ± 0.13 |
| | $ε$ | -7.63 ± 7.46 | 165 ± 12.2 | 0.76 ± 0.17 |
| | $η$ | 1.61 ± 3.63 | 80.0 ± 28.1 | 0.72 ± 0.36 |
| | $l_{eff}$ | -87.0 ± 93.9 | 1145 ± 125 | 0.88 ± 0.13 |
| $res^+$-Venus | $γ$ | 3.85 ± 2.60 | 13.6 ± 12.6 | 0.89 ± 0.14 |
| *N* = 12 flies | $δ$ | -4.91 ± 3.13 | 47.9 ± 12.1 | 0.93 ± 0.09 |
| | $ε$ | -3.19 ± 2.94 | 147 ± 13.7 | 0.77 ± 0.25 |
| | $η$ | 0.50 ± 2.83 | 87.8 ± 8.34 | 0.92 ± 0.07 |
| | $l_{eff}$ | -47.7 ± 25.2 | 958 ± 203 | 0.93 ± 0.08 |
| $res^+$-GFP | $γ$ | 3.43 ± 3.61 | 16.7 ± 14.5 | 0.92 ± 0.07 |
| *N* = 11 flies | $δ$ | -3.32 ± 2.82 | 55.1 ± 19.0 | 0.85 ± 0.12 |
| | $ε$ | -1.52 ± 1.43 | 160 ± 11.6 | 0.58 ± 0.30 |
| | $η$ | 0.33 ± 1.94 | 89.1 ± 9.39 | 0.72 ± 0.33 |
| | $l_{eff}$ | -32.4 ± 13.7 | 990 ± 233 | 0.93 ± 0.08 |
| $Res^-$ | $γ$ | 3.25 ± 4.18 | 19.7 ± 25.3 | 0.75 ± 0.29 |
| *N* = 12 flies | $δ$ | -4.37 ± 3.03 | 63.3 ± 13.5 | 0.74 ± 0.30 |
| | $ε$ | -2.99 ± 4.12 | 152 ± 16.3 | 0.57 ± 0.37 |
| | $η$ | -0.40 ± 3.71 | 96.1 ± 19.9 | 0.75 ± 0.32 |
| | $l_{eff}$ | -69.5 ± 37.6 | 1056 ± 167 | 0.89 ± 0.08 |

**Table S5** Linear regression fit statistics of leg angles and effective leg length ($l_{eff}$) at increasing force loading of the middle leg in *Drosophila* (body displacement 0.1 mm to 1.0 mm, N = 18 data points). *γ*, angle of coxo-trochanteral joint; *δ*, angle of femoro-tibial joint; *ε*, angle of tibio-tarsal joint; *η*, angle of end of tarsus and substrate. Angles are as defined in figures 1 and 6 of the main text. Outcome of statistical comparisons of means (ANOVA) is shown in Table S6. Data are represented as mean ± standard deviation. *Related to figure 6***.**

### Data S6: Linear regression fit statistics of leg angles and effective leg length

| Parameter | *p*-value slope | *p*-value offset | *p*-value $R^2$ |
|---|---|---|---|
| $\Gamma$ (°) | 0.19 | 0.75 | 0.08 |
| $\delta$ (°) | <0.002 $^{1,2;\ 2,3;\ 2,5;\ 2,6}$ | <0.001 $^{1,3;\ 2,3;\ 3,5;\ 1,6;\ 2,6;\ 5,6}$ | 0.01 $^{2,5}$ |
| $\varepsilon$ (°) | 0.07 $^{1,3}$ | <0.01 $^{2,5}$ | 0.40 |
| $\eta$ (°) | 0.34 | 0.27 | 0.40 |
| $l_{eff}$ *(mm)* | 0.02 $^{1,5}$ | 0.19 | 0.52 |

**Table S6.** ANOVA on linear regression fit statistics of leg joint angles and effective leg length (*cf.* Table S5). *p*-values > 0.05 suggest that the leg joint angles of the 6 tested fly strains are equal. Indices, Tukey post-hoc test results showing significant differences ($p < 0.05$) between strains; 1, *wildtype*; 2, *R104*; 3, *act5C*-cas9; 4 *res*$^+$-GFP; 5, *res*$^+$-Venus; 6 *res*$^-$. Data are represented fit statistics on mean values. *Related to figure 6*.

### Data S7: Stiffness (Young's modulus) and compliance

| *Fly strain* | Stiffness (µN mm$^{-1}$) | Compliance (mm µN$^{-1}$) | Young's modulus leg (kPa) | Young's modulus tendon (MPa) |
|---|---|---|---|---|
| *Wildtype* | 13.1 ± 7.97 | 0.11 ± 0.08 | 2.46 ± 1.66 | 1.31 ± 0.88 |
| *R104* | 11.5 ± 8.21 | 0.14 ± 0.08 | 1.75 ± 1.27 | 0.93 ± 0.67 |
| *act5C*-cas9 | 8.35 ± 7.59 | 0.25 ± 0.20 | 2.51 ± 1.58 | 1.34 ± 0.84 |
| *res*$^+$-GFP | 14.3 ± 7.94 | 0.10 ± 0.06 | 3.10 ± 1.84 | 1.65 ± 0.98 |
| *res*$^+$-Venus | 30.1 ± 23.2 | 0.05 ± 0.03 | 5.35 ± 5.36 | 2.85 ± 2.85 |
| *res*$^-$ | 7.75 ± 3.78 | 0.16 ± 0.07 | 1.29 ± 0.62 | 0.69 ± 0.33 |

**Table S7.** Stiffness, compliance and Young's modulus during force loading the middle leg of *Drosophila*. Values are calculated from linear regression fits to 14 loading values ($l_{eff}$ = 0.25 - 0.9 mm, Fig. 7D). Young's modulus is calculated for both mean outer diameter of the middle leg and mean diameter of the long leg tendon (see main text). N = 11 *wildtype* Canton S, N = 7 *R104*, N = 9 *act5C*-cas9, N = 11 *res*$^+$-Venus, N = 12 *res*$^+$-GFP, N = 15 *res*$^-$ flies. Data are represented as mean ± standard deviation. *Related to figures 7 and 8*.

### Data S8: Results from work-loop analysis

| *Fly strain* | Negative work (nJ) | Positive work (nJ) | Loss $E_{pot}$ (nJ) | Relative loss $E_{pot}$ (%) |
|---|---|---|---|---|
| *wildtype* | -0.71 ± 0.20 | 0.21 ± 0.13 | -0.50 ± | -71.1 ± 15.3 |
| *R104* | -0.59 ± 0.28 | 0.15 ± 0.08 | -0.44 ± | -74.3 ± 9.29 |
| *act5C*-cas9 | -0.47 ± 0.15 | 0.20 ± 0.09 | -0.28 ± | -57.9 ± 14.7 |
| *res*$^+$-GFP | -0.63 ± 0.18 | 0.15 ± 0.05 | -0.48 ± | -75.6 ± 5.81 |
| *res*$^+$-Venus | -0.57 ± 0.13 | 0.13 ± 0.06 | -0.45 ± | -78.2 ± 7.72 |
| *res*$^-$ | -0.56 ± 0.20 | 0.13 ± 0.06 | -0.44 ± | -76.1 ± 12.9 |

**Table S8.** Work-loop analysis during a complete force loading and unloading cycle of a middle leg. Negative work is the area under the loading curve ($l_{eff}$ = 0 - 1.0 mm, 20 data points, 0 - 10 s, see Fig. 7 of main text) and positive work the corresponding area during unloading. Areas with negative forces are considered. Loss $E_{pot}$, loss of elastic potential energy of the tested leg during unloading compared to loading sequence; relative loss $E_{pot}$, relative loss of elastic potential energy during unloading compared to loading sequence (100%). N = 11 *wildtype*, N = 7, *R104*, N = 9 *act5C*-cas9, N = 11 *res*$^+$-Venus, N = 12 *res*$^+$-GFP, N = 15 *res*$^-$ flies. Data are represented as mean ± standard deviation. *Related to figures 7 and 8*.

## Data S9: Statistics on leg properties

| *Fly strain 1* | *Fly strain 2* | *p*-value | | | | | |
|---|---|---|---|---|---|---|---|
| | | Stiffness and compliance | Young's modulus | Negative work | Positive work | Loss $E_{pot}$ | Relative loss $E_{pot}$ |
| *Wildtype* | *R104* | 0.57 | 0.27 | 0.11 | 0.20 | 0.22 | 0.40 |
| *Wildtype* | *act5C*-cas9 | 0.10 | 0.96 | <0.01 | 0.11 | <0.01 | 0.09 |
| *act5C*-cas9 | *R104* | 0.14 | 0.29 | 0.91 | 0.29 | 0.17 | 0.03 |
| *Wildtype* | *res*$^+$-GFP | 0.70 | 0.42 | 0.48 | 0.31 | 0.80 | 0.28 |
| *Wildtype* | *res*$^+$-Venus | 0.02 | 0.12 | 0.06 | 0.09 | 0.56 | 0.15 |
| *Wildtype* | *res*$^-$ | 0.04 | 0.06 | 0.06 | 0.09 | 0.23 | 0.23 |
| *res*$^+$-GFP | *res*$^-$ | 0.04* | 0.001 | 0.42 | 0.32 | 0.45 | 0.49 |
| *res*$^+$-GFP | *res*$^+$-Venus | 0.03* | 0.37 | 0.37 | 0.36 | 0.75 | 0.31 |
| *res*$^+$-Venus | *res*$^-$ | < 0.001 | < 0.001 | 0.68 | 0.80 | 0.44 | 0.72 |

**Table S9.** Statistical comparison of leg properties. Data are tested on normality using as Shapiro-Wilk test and data differences using a Mann-Whitney-U test. *, in case of normality we used a *t*-test for data comparison. Data are represented as mean as shown in Tables S7 and S8. *Related to figures 7 and 8.*

## Methods S10: Calibration of experimental setup

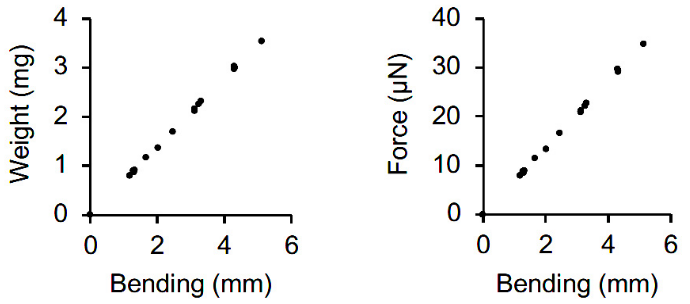


**Figure S10**. Calibration of tungsten sensor wire with predefined weights (left; linear regression, y=0.697x - 0.017) or corresponding force (right; linear regression, y=6.83x - 0.165) plotted against wire deflection at tarsal-wire contact point. Data are represented as mean. *Related to figure 1.*
